# Impaired Ability to Suppress Excitability of Antagonist Motoneurons at Onset of Dorsiflexion in Adults with Cerebral Palsy

**DOI:** 10.1155/2018/1265143

**Published:** 2018-10-09

**Authors:** Svend Sparre Geertsen, Henrik Kirk, Jens Bo Nielsen

**Affiliations:** ^1^Department of Nutrition, Exercise and Sports, University of Copenhagen, Copenhagen, Denmark; ^2^Department of Neuroscience, University of Copenhagen, Copenhagen, Denmark; ^3^Helene Elsass Center, Charlottenlund, Denmark

## Abstract

We recently showed that impaired gait function in adults with cerebral palsy (CP) is associated with reduced rate of force development in ankle dorsiflexors. Here, we explore potential mechanisms. We investigated the suppression of antagonist excitability, calculated as the amount of soleus H-reflex depression at the onset of ankle dorsiflexion compared to rest, in 24 adults with CP (34.3 years, range 18–57; GMFCS 1.95, range 1–3) and 15 healthy, age-matched controls. Furthermore, the central common drive to dorsiflexor motoneurons during a static contraction in the two groups was examined by coherence analyses. The H-reflex was significantly reduced by 37% at the onset of dorsiflexion compared to rest in healthy adults (*P* < 0.001) but unchanged in adults with CP (*P* = 0.91). Also, the adults with CP had significantly less coherence. These findings suggest that the ability to suppress antagonist motoneuronal excitability at movement onset is impaired and that the central common drive during static contractions is reduced in adults with CP.

## 1. Introduction

When we move, our nervous system ensures that our muscles are activated to the appropriate extent and at the right time in relation to each other so that the movement may progress according to our intentions and with little or no conscious attention required. This is not something that comes easily and quickly. It takes children 10–12 years to attain the mature characteristics of bipedal gait seen in adults [1–4]. Step-to-step variability of gait is significantly larger than in adults and involves significantly more coactivation of antagonistic muscles [1, 3, 4]. Reaching and grasping follow a similar developmental trajectory, and an adult-like movement pattern is not achieved until around 12–14 years of age [5].

People with early brain lesion (cerebral palsy (CP)) in contrast continue to show very significant coactivation of muscles and high step-to-step variability of gait into adulthood [6–8]. They also lack the normal maturation of gating of sensory feedback at rest [9] and during gait [10, 11]. This may possibly be linked to an impaired development of the ability to predict and therefore suppress sensory feedback, which is linked to adequate prediction of the sensory consequences of the movement [12]. However, little is known about the underlying neural mechanisms that are responsible for the maintained coactivation pattern in adults with CP.

One of the mechanisms known to be important for the coordination of antagonist muscles is Ia reciprocal inhibition. Ia reciprocal inhibition involves a group of interneurons, which are activated through collaterals from descending pathways in parallel with agonist motoneurons and project to antagonist motoneurons [13]. In contrast to what is usually observed in other people with lesion of descending motor pathways, such as stroke and multiple sclerosis [14, 15], Ia reciprocal inhibition appears to be similar at rest in adults with CP as in healthy, age-matched controls [9]. However, pathophysiological changes in transmission in spinal motor circuitries observed at rest may have little relevance for how those circuitries are controlled and modulated during motor activities [16–18]. Indeed, Leonard et al. [19] found that Ia reciprocal inhibition was similar in adults with CP and in healthy adults when measured at rest, but during static agonist contraction, the inhibition was increased in healthy subjects and reduced in adults with CP [19]. Morita et al. have also shown impaired regulation of Ia inhibition at the onset of agonist contraction in adults with multiple sclerosis and suggested that this could explain increased coactivation of antagonists in these subjects [20]. We recently showed that impaired gait function in adults with CP is associated with the ability to perform fast ankle movements [21], but it is not known how reciprocal inhibition is modulated at the onset of contraction in adults with CP.

Here, we consequently hypothesized that impaired descending control of spinal inhibitory circuits is responsible for the inability of adults with CP to adequately suppress antagonist muscle activity in relation to voluntary movement and that this may explain their continued coactivation during functional motor tasks. To assess modulation of spinal reciprocal inhibition, we measured the suppression of the soleus H-reflex at the onset of dorsiflexion, and to assess the central drive to the agonist motor pool (dorsiflexors), we measured the size of coupled oscillations in tibialis anterior motor units.

## 2. Material and Methods

### 2.1. Participants

Twenty-four adults diagnosed with CP (age 34.3 years, range 18–57; 15 men, 9 women; GMFCS 1.95, range 1–3) were recruited through the Danish Cerebral Palsy Organization. Fifteen subjects were diplegic, eight hemiplegic, and one quadriplegic. All subjects were described as spastic, and most of the subjects had received antispastic medication for shorter or longer periods. Many subjects had a history of multiple surgeries. See [21] for a detailed description of the participants. Furthermore, 15 age-matched (age 32.9 years, range 23–47; 9 men, 6 women) neurologically healthy adults were recruited to serve as a healthy control group.

The study was approved by the local ethics committee (H-4-2012-107), and all procedures were conducted within the standards of the Helsinki declaration. Prior to the experiments, all the participants received written and verbal information, and a consent form for participation was obtained.

### 2.2. Testing Procedures

Functional reciprocal inhibition (experiment 1) and central common drive (experiment 2) were assessed on the same day following the application of electromyography (EMG) electrodes and tests of the maximal voluntary contraction strength (MVC) and the rate of force development (RFD).

#### 2.2.1. EMG Recordings

EMG activity was recorded using bipolar electrodes (Ambu Blue sensor N-10-A/25, Ambu A/S Ballerup; recording area 0.5 cm^2^, interelectrode distance 2 cm) placed over the soleus muscle and the proximal and distal parts of the tibialis anterior muscle (TA_prox_ and TA_dist_, respectively). The skin was gently abraded with sandpaper (3M red dot; 3M, Glostrup, Denmark). A ground electrode was placed on the distal part of the tibia. EMG signals were filtered (band-pass, 5 Hz–1 kHz), amplified (500-2000x), sampled at 2 kHz, and stored on a PC for offline analysis.

All EMG and H-reflex measurements (see below) were normalized to the maximal M-response (*M*_max_) evoked in either the TA or soleus muscle by supramaximal stimulation (1 ms rectangular pulses; model DS7A, Digitimer, Hertfordshire, UK) of the common peroneal nerve or the tibial nerve, respectively. In these measurements, the intensity of stimulation of the respective nerves was increased from a subliminal level until there was no further increase in the peak-to-peak amplitude of the M-response with increasing stimulation intensity [22].

#### 2.2.2. Measurement of MVC, RFD, and Cocontraction

The MVC and RFD procedures have been comprehensively described by Geertsen et al. [21]. Briefly, subjects were seated in a chair with their leg fastened to a stationary dynamometer and were carefully instructed to contract “as fast and forcefully as possible” and to hold the contraction for about 3 seconds. During each trial, the subject was verbally encouraged by the experimenter to produce maximal torque. Each subject performed 3 dorsiflexions with maximal effort. If an initial countermovement (identified by a visible drop in the torque trace) was observed, a new trial was performed. Data was recorded with Spike 2.611 software (CED 1401+; Cambridge Electronics Design, Cambridge, UK). Offline, the trial that produced the highest dorsiflexion peak torque (MVC_DF_) was determined. The MVC_DF_ trial was then used to calculate the RFD at 200 ms following the onset of contraction (RFD_200_) as a measure of explosive muscle force. The level of cocontraction in the MVC_DF_ trial was calculated as the area of rectified, smoothed soleus EMG (in percent of soleus *M*_max_) divided by the area of rectified, smoothed TA_dist_ EMG (in percent of *M*_max_ in TA_dist_) for the first 1000 ms following the onset of TA_dist_ EMG.

#### 2.2.3. Experiment 1: Functional Reciprocal Inhibition

Functional reciprocal inhibition was evaluated by comparing the size of soleus H-reflexes at rest with H-reflexes elicited at the onset of explosive dorsiflexion contractions. H-reflexes were elicited by stimulation (1 ms rectangular pulses; model DS7A, Digitimer, Hertfordshire, UK) of the tibial nerve using a ball-shaped monopolar electrode (Simon electrode) placed in the popliteal fossa and the anode placed proximal to the patella. All H-reflex measurements were normalized to *M*_max_.

**Figure 1:**
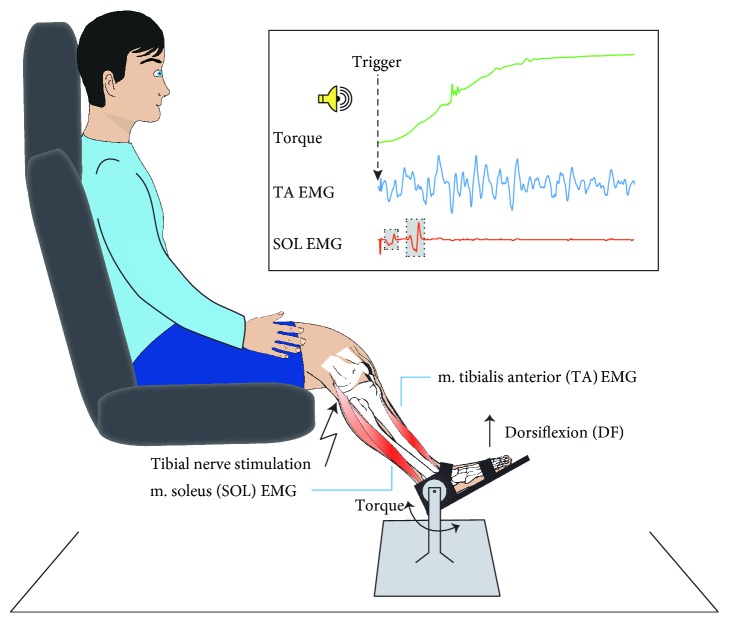
Experimental setup. Subjects were seated with their examined leg fastened to a stationary dynamometer. For experiment 1, subjects were instructed to dorsiflex their foot as fast as possible to 50% of MVC in response to an auditory cue. A window discriminator made it possible to time the tibial nerve stimulation to the onset of tibialis anterior (TA) EMG activity. This elicited an M-response (first grey shaded box) and an H-reflex (second grey shaded box) in the soleus (SOL) EMG. At least 45 trials, 15 tibial nerve stimulation and 30 no stimulation trials randomly interspersed, were obtained during dorsiflexion contraction. In experiment 2, subjects were asked to keep a steady dorsiflexion contraction at 10% of MVC for 2 min while given visual feedback of the target torque.

To produce comparable afferent input to the soleus motoneuron pool at rest and at the onset of dorsiflexion contraction, the tibial nerve stimulation intensity was adjusted, if necessary, to elicit an M-response of approximately 10% of *M*_max_ in all trials. However, the actual intensities used were similar at rest (15.78 ± 4.86 mA) and at the onset of dorsiflexion (15.64 ± 4.83 mA). At rest, 15 H-reflexes were elicited with an interstimulus interval of 10 s. The subject was then asked to dorsiflex the ankle as fast as possible every 10 s (to 50% of MVC_DF_ to avoid fatigue) following an auditory cue (see Figure 1). A window discriminator made it possible to time the tibial nerve stimulation to the onset of TA EMG activity.

**Figure 2:**
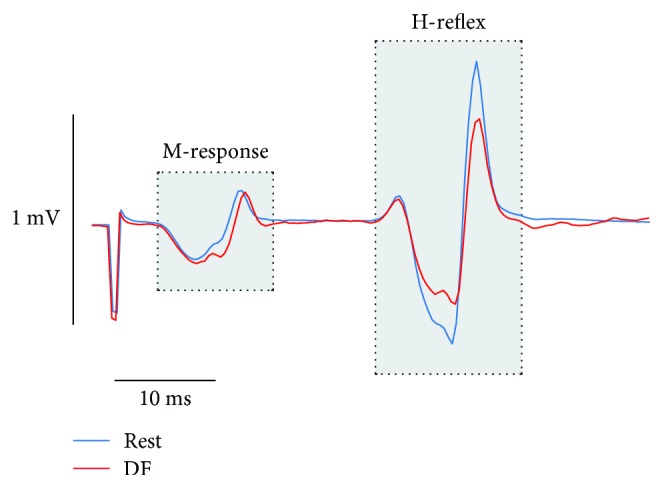
Evaluation of functional reciprocal inhibition in a healthy control. To produce a comparable afferent input to the soleus motoneuron pool at rest and during contraction, the tibial nerve stimulation intensity was adjusted to keep the M-response (first shaded box) at approximately 10% of *M*_max_ both at rest and at the onset of dorsiflexion (DF). The peak-to-peak amplitude of the H-reflex (second shaded box) could then be compared in the two situations, as a measure of the ability to suppress excitability of antagonist motoneurons at the onset of dorsiflexion (i.e., functional reciprocal inhibition).

At least 45 trials, 15 tibial nerve stimulation and 30 no stimulation trials randomly interspersed, were obtained during dorsiflexion contraction. Offline, the peak-to-peak amplitude of the H-reflex at the onset of dorsiflexion was then compared to rest (see Figure 2).

In six participants with CP and one participant from the healthy control group, it was not possible to obtain an H-reflex at rest while keeping the M-response at 10% of *M*_max_. Also, two participants with CP could not produce a voluntary dorsiflexion contraction. These subjects were therefore excluded from this part of the analyses.

#### 2.2.4. Experiment 2: Central Common Drive

The common drive to the dorsiflexor motoneuron pool was evaluated by coherence analysis of the surface EMG activity from TA_prox_ and TA_dist_ obtained while subjects performed a static dorsiflexion contraction to a torque level of 10% MVC_DF_ for two minutes while given visual feedback. Coherence in the beta band (15–35 Hz) has been shown to be dependent on intact corticospinal activity [23–25] and is therefore thought to reflect central common drive.

**Figure 3:**
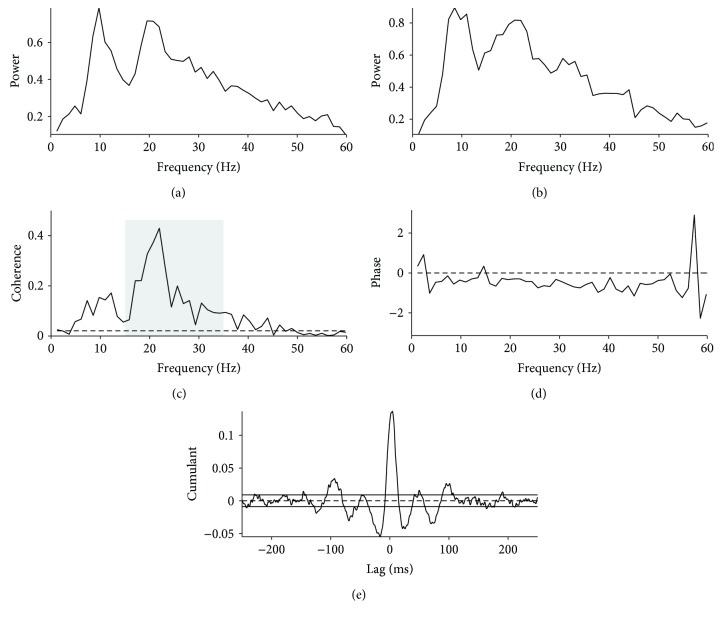
Example of intramuscular coherence analyses from a healthy control. (a, b) Autospectra from the proximal (TA_prox_) and the distal (TA_dist_) parts of the tibialis anterior during static dorsiflexion. (c) Coherence at frequencies from 1 to 60 Hz between TA_prox_ and TA_dist_ rectified EMG signals. The dashed horizontal line denotes the upper 95% confidence level, and the grey shaded area highlights the 15–35 Hz frequency band referred to as beta coherence. (d) The phase between the TA_prox_ and TA_dist_ rectified EMG signals indicating the synchronization between coherent EMG frequencies. (e) Cumulant density (range ± 250 ms) associated with the coherence.

Time and frequency domain analysis of the data was performed in MATLAB (version R2016b, MathWorks, MA, USA) using the methods described by Halliday et al. [26] and Farmer et al. [27]. Full-wave rectification of surface EMG signals was performed in order to maximize the information regarding timing of motor unit action potentials while suppressing information regarding waveform shape [28, 29]. The two rectified TA EMG signals were then normalized to have unit variance [30]. Rectified and normalized EMG signals are assumed to be realizations of stationary zero mean time series, denoted by *x* and *y*. The analysis of individual records generated estimates of the autospectra of the two EMGs [*fxx*(*λ*), *fyy*(*λ*)], and their cross-spectra [*fxy*(*λ*)]. Frequency domain analyses were performed with a frequency resolution of 1 Hz. We estimated three functions that characterize the signals' correlation structure: coherence, *R*_*xy*_(*λ*)^2^; phase, Φ_*xy*_(*λ*); and cumulant density, *q*_xy_(*u*). Coherence describes the linear association between two signals at each frequency of interest and reflects the consistency of phase differences and amplitude ratios between signals across trials. Coherence estimates are bounded measures of association defined over the range of [0, 1] where 0 indicates no association between signals, and 1 indicates a strong association; cumulant density estimates are not bounded, and phase is defined over the range [−*π*, +*π*]. For the present data, coherence estimates provide a measure of the fraction of the activity in one surface EMG signal (TA_prox_) that is correlated with the activity in the second surface EMG signal (TA_dist_). In this way, coherence estimates quantify the strength and range of frequencies of common rhythmic synaptic inputs distributed across the motoneuron pool [27, 31–33]. The timing relations between the EMG signals are estimated from the phase. The cumulant density provides a time-domain representation of the correlation structure analogous to the cross-correlogram. The significance of the individual coherence and cumulant density estimates are assessed by inclusion of an upper 95% confidence limit in coherence plots and upper and lower 95% confidence limits in cumulant density plots (see example in Figure 3), based on the assumption of statistical independence. For details, see [26].

All individual coherence plots were visually inspected for signs of cross-talk, i.e., high coherence across a wide range of frequencies and close to zero lag synchronization in the time domain as evidenced in cumulant density plots [27]. None of the coherence plots displayed these characteristics, so all data from each group were pooled resulting in single group estimates at each frequency of interest for the adults with CP and the healthy controls, respectively. Pooled coherence estimates, like individual coherence estimates, provide a normative measure of linear association on a scale from 0 to 1 [30]. The interpretation of pooled estimates is similar to those for individual records, except that any interference relates to the population as a whole [27]. Group differences were investigated using the *χ*^2^ extended difference of coherence test [34], a nonparametric test that provides the amount of pooled coherence differences between groups at each frequency in relation to an upper 95% confidence interval limit.

As described previously, two participants with CP could not produce a voluntary dorsiflexion contraction. These subjects were therefore excluded from this part of the analyses.

#### 2.2.5. Statistics

Sigma Plot statistical software version 12.5 was used for statistical analysis. A one-way ANOVA was used to investigate differences in the amount of cocontraction between adults with CP and healthy controls. A two-way repeated measure ANOVA with group (CP or CON) and state (rest or onset of dorsiflexion) was applied for H-reflex and M-response analyses. To investigate possible associations between H-reflex modulation and muscle strength in the adults with CP, we used the Pearson product-moment correlations. For experiment 2, the extended *χ*^2^ test was used to calculate the difference of coherence between adults with CP and healthy controls. Coherence was also quantified as the sum (i.e., area) of alpha (5–15 Hz) and beta (15–35 Hz) coherence. These values were transformed logarithmically to symmetrize distributions for statistical analyses [35] and compared using Student's *t*-test. Associations between H-reflex modulation and coherence area within and across groups were assessed by means of the Pearson product-moment correlations. Statistical significance was given for *P* values smaller than 0.05. Data are presented as the means ± standard error unless reported otherwise.

## 3. Results

Data from the test of the dorsiflexion strength has already been reported by Geertsen et al., where we showed that for adults with CP, MVC_DF_ was 42% of healthy controls (*P* < 0.001) and RFD_200_ only 21% healthy controls (*P* < 0.001) [21]. Further analyses performed here showed that during MVC_DF_, adults with CP exhibited significantly more cocontraction (10.6 ± 1.5%) than healthy controls (5.9 ± 1.2%; *P* = 0.003).

### 3.1. Functional Reciprocal Inhibition

**Figure 4:**
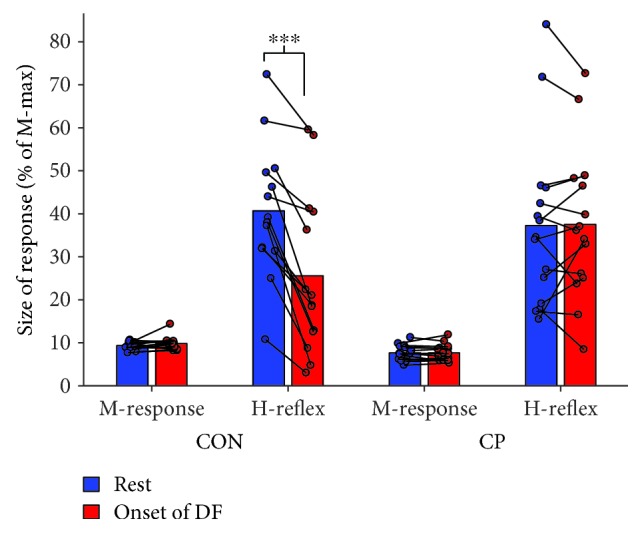
Functional reciprocal inhibition in healthy controls and adults with CP. Mean and individual M-response and H-reflex amplitudes in % of the maximal M-responses (M-max) at rest and at the onset of dorsiflexion (DF). The M-response was comparable at rest and at the onset of DF for both healthy controls (CON) and adults with CP. At the onset of DF, the H-reflex was significantly reduced in the healthy controls, whereas it was unchanged in adults with CP. Significant differences between rest and onset of DF are indicated by ^∗∗∗^*P* < 0.001.

We found a significant group-state interaction when comparing the H-reflex amplitude at rest with the amplitude at the onset of dorsiflexion for the adults with CP and healthy controls (*F*_1,27_ = 22.35, *P* < 0.001). Post hoc analysis revealed that the healthy control group significantly reduced the H-reflex amplitude by 37% from 40.7 ± 4.1% of *M*_max_ at rest to 25.6 ± 5.0% at the onset of contraction (*P* < 0.001). This functional reciprocal inhibition was not evident in the participants with CP (rest: 37.3 ± 5.1%, dorsiflexion: 37.5 ± 4.5%, *P* = 0.91; Figure 4). There was no significant group-state interaction when comparing the amplitude of the M-response at rest with the amplitude at the onset of dorsiflexion for the adults with CP and healthy controls (*F*_1,27_ = 1.32, *P* = 0.26), indicating a comparable afferent input to the soleus motoneuron pool in the two states for both groups (Figure 4).

In adults with CP, the amount of H-reflex suppression was significantly correlated with both MVC_DF_ (*r* = 0.58, *P* = 0.02) and RFD_200_ (*r* = 0.56, *P* = 0.03).

### 3.2. Central Common Drive

Figure 3 shows individual coherence data from a healthy control during static dorsiflexion. The autospectra for TA_prox_ and TA_dist_ (Figures 3(a) and 3(b)) illustrate the origin of the elements used for time and frequency domain analysis. Coherence estimates calculated from the autospectra and cross-spectra are shown in Figure 3(c). Here, a clear peak can be seen in the beta (15–35 Hz) frequency band, as well as a small peak in the alpha (5–15 Hz) frequency band.  Figure 3(d) shows the phase difference between the two rectified EMGs. The cumulant density constructed from the rectified EMG data is shown in Figure 3(e). Note the clear central peak around 0 ms indicating synchronization between the rectified EMG data from TA_prox_ and TA_dist_.

**Figure 5:**
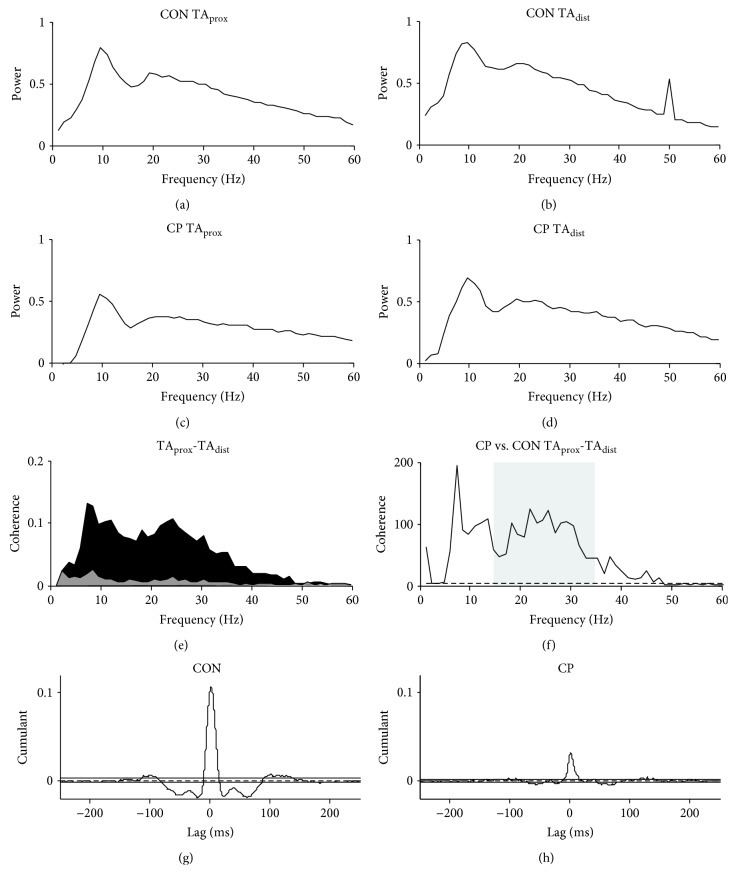
Pooled coherence plots and *χ*^2^ analyses. (a–d) Pooled power in the proximal (TA_prox_) and distal (TA_dist_) parts of the tibialis anterior during static dorsiflexion for the healthy control group (CON) and adults with cerebral palsy (CP). (e) Pooled coherence between TA_prox_ and TA_dist_ for adults with CP (grey) and CON (black). (f) *χ*^2^ analyses of the difference between adults with CP and the CON group. (g–h) Pooled cumulant density associated with the coherence for the CON and the CP groups.

Pooled TA-TA EMG coherence estimates from adults with CP and healthy controls are presented in Figure 5(a). For both groups, pooled alpha and beta coherence estimates exceeded significance levels, but adults with CP displayed considerably less coherence across all frequencies compared with the healthy adults. This observation was confirmed by the results from the extended *χ*^2^ test of the group coherence estimates displayed in Figure 5(b), which showed a statistical difference at both alpha (5–15 Hz) and beta (15–35 Hz) frequencies.

**Figure 6:**
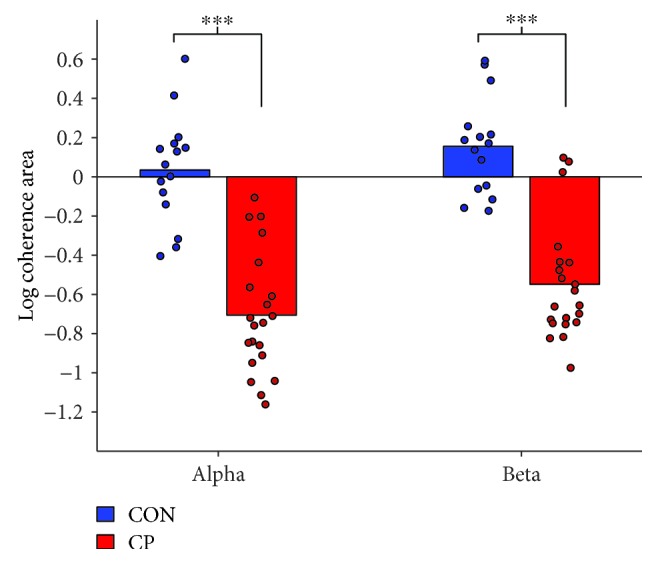
Coherence area estimates. Logarithmic coherence area between the proximal (TA_prox_) and distal (TA_dist_) part of the tibialis anterior in alpha (5–15 Hz) and beta (15–35 Hz) frequencies during static dorsiflexion for healthy controls (CON; blue) and adults with CP (red). Significant differences between the groups are indicated by ^∗∗∗^*P* < 0.001.

Reduced TA-TA coherence in adults with CP was also confirmed when comparing the coherence areas at alpha and beta frequencies across individuals (Figure 6). Compared to healthy controls, adults with CP had significantly less alpha (−0.705 ± 0.083 vs. 0.034 ± 0.071; *P* < 0.001) and beta (−0.549 ± 0.079 vs. 0.155 ± 0.064; *P* < 0.001) coherence.

We also investigated possible associations between the central common drive to the TA motoneuron pool (log TA-TA coherence area) and the ability to suppress the antagonist motoneuron pool (reduction in soleus H-reflex amplitude at the onset of DF compared to rest). We observed a negative correlation (i.e., more coherence and larger H-reflex reduction) that approached significance in both healthy controls (*r* = −0.53, *P* = 0.05) and adults with CP (*r* = −0.43, *P* = 0.13), and the correlation was significant across groups (*r* = −0.74, *P* < 0.001).

## 4. Discussion

The main findings of this study are that the central common drive to ankle dorsiflexors and functional reciprocal inhibition of ankle plantar flexors are impaired in adults with CP. This may contribute to the reduced coordination of antagonistic muscles and impaired gait function observed in adults with CP.

### 4.1. Impaired Functional Reciprocal Inhibition in Adults with CP

The inability of adults with CP to suppress the soleus H-reflex to the same extent as healthy adults at the onset of dorsiflexion is similar to what has been observed in adults who have acquired lesion of descending motor pathways as adults because of multiple sclerosis [20], stroke [36], or spinal cord injury [37, 38]. Our findings indicate that a similar impaired control in adults may also be seen as the result of a lesion early in life and that the intervening years of motor practice and experience apparently do little to change this. We were not able to address the mechanisms responsible for the reduced suppression of the H-reflex further, but based on previous experiments in healthy subjects [13] and adults with lesion of central motor pathways [20, 39], it appears likely that impaired regulation of spinal interneurons responsible for conveying reciprocal Ia inhibition and/or presynaptic inhibition of Ia afferents is involved. These two spinal interneuronal populations have been shown to be responsible for suppressing stretch reflex activity in antagonist muscles by reducing antagonist motoneuronal excitability and limiting the input from antagonist stretch-sensitive receptors to the motoneurons at the onset of movement [39, 40]. Suppressing transmission in the stretch reflex circuitry through two different populations of interneurons and at two different points may be an efficient safeguard to ensure that stretch of the antagonists does not elicit unwanted stretch reflex activity.

It follows from this that the impaired functional reciprocal inhibition that we have found in adults with CP here could provide an explanation of the inability of the subjects to generate force quickly and efficiently [21]. Previous studies have indicated that people with central motor lesions may move slowly in order to avoid eliciting stretch reflex activity in antagonists when they are stretched at the onset of (fast) movements [15, 16, 41, 42]. However, some caution is required. The adults with CP in our study did not as a group have larger stretch reflexes than healthy subjects at rest (see Figure 4) and the subjects who were the least able to suppress the H-reflex were not those who had the largest stretch reflexes. It should also be kept in mind that the opposite causal relationship is equally likely and that H-reflexes were only slightly suppressed in the adults with CP because they were unable to generate an efficient descending drive to the agonist motoneurons and thereby activate reciprocal inhibitory mechanism efficiently. Our observations of strongly reduced common synaptic drive to ankle dorsiflexors support this interpretation.

### 4.2. Reduced Central Common Drive in Adults with CP

We used coherence between surface EMG recordings obtained from two different sites over the tibialis anterior muscle as a measure of the central common drive to populations of motoneurons within the same motor pool. This approach requires that the EMG recordings reflected the activity of different populations of motoneurons and that the recordings were not contaminated by cross-talk. Although cross-talk is difficult to rule out definitively, we are confident that we were able to minimize cross-talk to the extent that it cannot explain the findings in the present study: First, we made sure always to position electrodes at least 10 cm apart since muscle fibers in the tibialis anterior muscle have been shown not to exceed 6 cm [43, 44]. Second, cross-talk is easily identified from coherence between the recordings at all frequencies and a large, narrow peak at zero time lag in the cumulant density function [45, 46]. The recordings in the present study only showed coherence within restricted frequency bands (Figures 3 and 5) and peaks of synchronization in the cumulant density function were always found to have a distinct lag with respect to zero. We may therefore safely conclude that the observed coherence and synchronization peaks in the cumulant density function reflect a common central drive to the tibialis anterior motoneurons in the spinal cord [47–49]. The narrow central peak in the cumulant density function and the coherence dominantly in the alpha and beta bands are similar to what has been observed in numerous studies during static contraction in healthy adults previously [31, 47, 49]. There are strong arguments supporting the notion that the narrow central synchronization reflects input to the motoneurons from collaterals of common last order neurons [31, 47, 49], and there is also strong evidence to suggest that the coherence in the beta band reflects activity in corticospinal neurons and that the central drive responsible for these two phenomena therefore originates in the motor cortex and may possibly be explained by activity in the direct monosynaptic corticospinal pathway to the spinal motoneurons [31, 47, 49]. If so, our findings would be consistent with impaired transmission in the corticomotoneuronal pathway in adults with CP, since both coherence and the central short-term synchronization peak in the cumulant density function were reduced in this group. Similar findings with similar interpretation have been reported previously for children with CP [3, 7] and for adults with spinal cord injury [45], stroke [23, 25], and multiple sclerosis [50], but we believe that our findings are the first to demonstrate this for adults with CP. Although the coherence measurements were performed during static contraction rather at the onset of dorsiflexion where reciprocal inhibition was evaluated, the two measures were correlated, and it makes sense from a physiological perspective that the reduced common drive to the dorsiflexors and the impaired reciprocal inhibition at the onset of dorsiflexion are related. The corticomotoneuronal pathway has been shown to be important for the initiation of fast, ballistic movements such as the dorsiflexion we used when testing reciprocal inhibition [51–53]. Corticomotoneuronal cells have also been shown to have collaterals to the Ia inhibitory interneurons responsible for reciprocal inhibition [54–56]. It is therefore, in our opinion, very likely that the reduced coherence between tibialis anterior motor units during static dorsiflexion and the reduced reciprocal inhibition at the onset of dorsiflexion are both linked to impaired transmission in the corticomotoneuronal pathway in the adults with CP.

Petersen et al. [3] found that coherence between tibialis anterior motor units during both static dorsiflexion and gait reached adult levels when children are 10–12 years old in parallel with reduced step-to-step variability of gait and suggested that this was related to the development of the corticospinal tract. In children with CP, this development of coherence was not observed and Petersen et al. [7] therefore suggested that the development of corticospinal drive was impaired. We may now extend these findings to conclude that adults with CP continue to show impaired corticospinal drive to the dorsiflexors and that this also impacts the coordination of antagonistic muscles. It follows that the intervening years of motor practice have not been sufficient to change this.

In children younger than 10 years, 4 weeks of daily treadmill training may increase coherence between tibialis anterior motor units in parallel with improved ability to lift the toes and make ground contact with the heel during gait [57]. This suggests that transmission in the corticospinal pathway is sufficiently plastic in this age group to induce important functional improvements through relatively short-lasting training. However, Willerslev-Olsen et al. [57] also found that such improvements were not found in children older than 10 years and it may therefore be anticipated that this is also the case in adults, although we have at present no knowledge about this. This may be put into the context of current ideas in computational neuroscience, which suggests that motor abilities are the result of a continuous updating of a predictive model that monitors the discrepancy between predicted and actual sensory consequences of movement [58–60]. With 10–12 years of gait experience, a relatively precise predictive model is likely to have been developed and it may therefore be more difficult to alter and require more training than earlier in life. This is consistent with the findings showing that an adult-like gait pattern with little variability (and little cocontraction) is attained around 10–12 years of age [1–4]. It is of interest in this relation that impedance control (i.e., cocontraction of antagonists) and slow movements (i.e., low RFD) have been found to be an optimal control strategy under dynamic conditions that are difficult to predict [61–63]. The characteristics of gait and other movements in adults with CP thus may reflect the most optimal strategy that their nervous system could find under the restrictions imposed by weak muscles and noisy and relatively unpredictable sensory feedback signals. It follows from this that efficient interventions in this group will have to involve “de-learning” of the unwanted movement pattern (cocontraction). This may be followed by learning of a more adequate movement pattern once the prerequisites for this have been established by strengthening muscles, reducing noise in the motor and sensory systems and facilitating relevant sensory signals.

## 5. Conclusion

We have shown in this study that the central common drive to ankle dorsiflexors and functional reciprocal inhibition of ankle plantar flexors are impaired in adults with CP. This likely reflects the most optimal control strategy under the constraints imposed by an early brain lesion. We suggest that the development of efficient functional interventions in adults with CP will have to take into account that all movements—including “abnormal” movements—may have to be seen as the result of a long learning process involving predictive coding of the sensory consequences of movement.

## Data Availability

All data files will be available from the https://zenodo.org/ database.
